# Laparoscopic Repair of a Large Duodenal Perforation Secondary to an Indwelling Nasogastric Tube in a Tracheotomized Adult

**DOI:** 10.1155/2013/503151

**Published:** 2013-03-23

**Authors:** Sanoop Koshy Zachariah

**Affiliations:** Department of General, Laparoscopic and Gastrointestinal Surgery, MOSC Medical College Kolenchery, Cochin 682311, India

## Abstract

Laparoscopic repair of perforated duodenal ulcers is safe and effective in centers with experience and increasingly performed by laparoscopic surgeons. However, the role of laparoscopy for the management of large duodenal perforations (>1 cm) is unclear. To date, no experience has been reported with emergency laparoscopic repair of large perforations for gastroduodenal ulcers. The commonest reason for conversion to open surgery is a perforation size of more than 1 cm. This paper reports a case of a large duodenal perforation due to a nasogastric tube in a 26-year-old male who had undergone a tracheostomy, following a cut-throat injury. This large perforation was successfully diagnosed and repaired laparoscopically. This is probably the first paper in the English literature to report duodenal perforation due to a nasogastric tube in an adult and also the first report of a successful laparoscopic repair of a large duodenal perforation.

## 1. Introduction

Laparoscopic repair of perforated duodenal ulcers is safe and effective in centers with experience and increasingly performed by laparoscopic surgeons. However, based on the existing literature, it is uncertain whether large duodenal perforations have been managed laparoscopically. Studies have shown that the commonest reasons for conversion from laparoscopic to open surgery is the finding of a large perforation (>1 cm) [1]. A consensus conference recently reported that laparoscopic repair of perforated gastric and duodenal ulcers is safe and effective in centers with experience, and to date no experience has been reported with emergency laparoscopic repair of large perforations [2]. In all these studies analyzed for the laparoscopic technique, the patients had small ulcers (mean diameter of 1 cm) and all the patients received simple suture, mostly with omental patch, or suture-less repair.

Duodenal perforations due to nasoenteral tubes are a recognized complication in pediatric patients [3, 4]. The present paper reports a case of a large duodenal perforation in a tracheotomiced adult, caused by an indwelling feeding nasogastric tube, which was managed laparoscopically. The paper discusses the potential complications of gastrointestinal intubation and also diagnostic role of laparoscopy in such situations and its possibility in management of large duodenal perforations.

## 2. Case Report and Operative Technique

A 26-year-old male had sustained a partial transverse tracheal transection following a cut-throat assault using a knife. There were no other significant findings on clinical examination and the abdomen appeared to be normal. The patient was initially managed by the “otorhinolaryngology team.” He underwent a neck exploration, followed by a primary suture repair of tracheal transection and a tracheostomy was also performed. A flexible polyvinyl nasogastric tube (14 Fr) was instituted for the purpose of enteral feeding. The patient also received intravenous antibiotics and proton pump inhibitors. The patient received feeds and seemed to be recuperating well until on the fifth POD (postoperative day) when he developed severe upper abdominal pain and distension with clinical features of peritonitis. The patient had no previous history suggestive of acid peptic disease. Laboratory investigations revealed borderline leucocytosis with elevated polymorphs, normal serum amylase, and lipase values. Plain erect abdominal radiograph was inconclusive. Ultrasonography revealed moderate intraperitoneal free fluid with dilated bowel loops. The patient was taken up for emergency diagnostic laparoscopy under general anesthesia.

**Figure 1:**
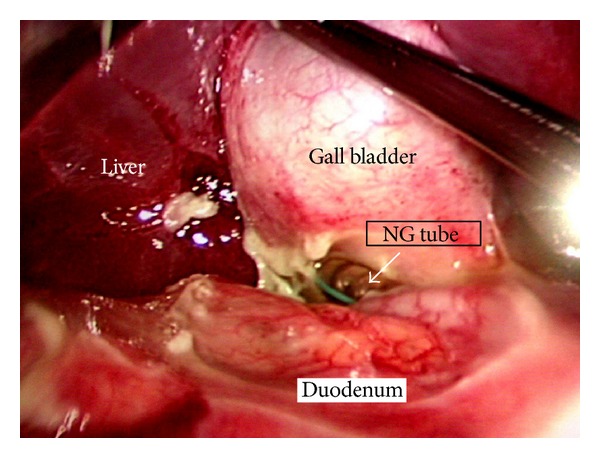
The nasogastric (NG) tube (white arrow) can be seen perforating the duodenum and impacting on the gall bladder.

**Figure 2:**
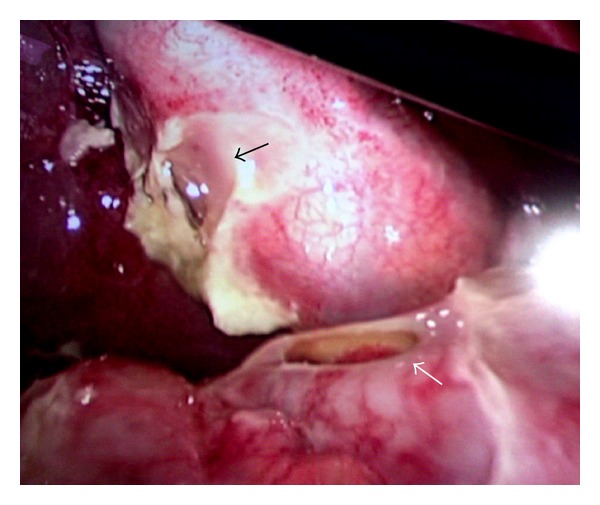
The large duodenal perforation (white arrow) is clearly seen after withdrawing the NG tube. The site of impaction of the NG tube on the gall bladder is also seen (black arrow).

**Figure 3:**
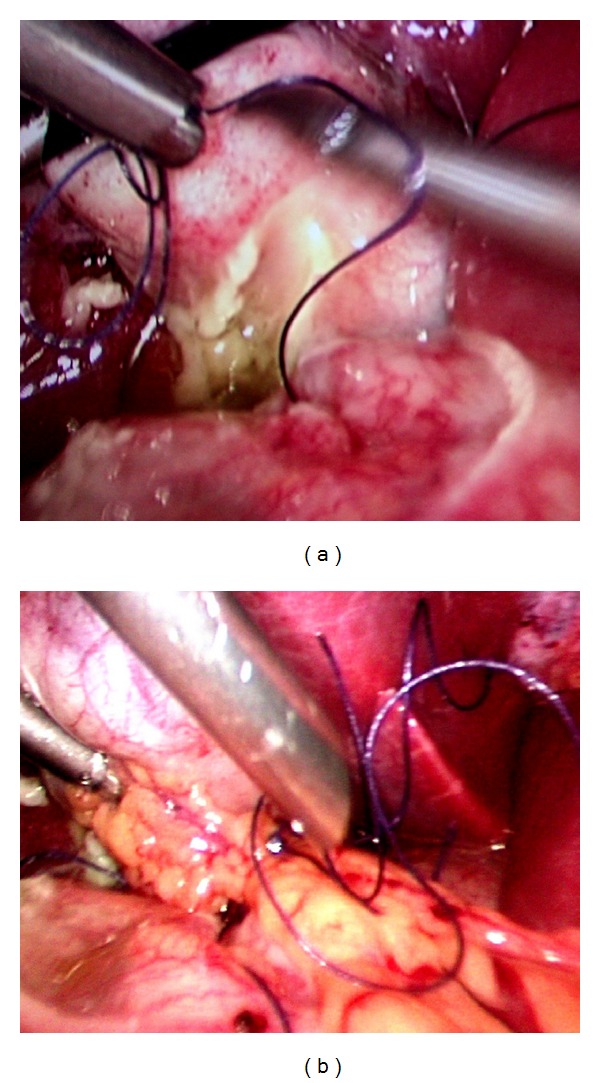
Repair of the large ulcer by primary suture (a) followed by onlay pedicled omentoplasty (b).

The open technique of laparoscopic access was used. Three ports, namely, a 10 mm (umbilical port for the 30° videoscope) and two 5 mm ports in the right and left midclavicular line were used (working instruments). Laparoscopic evaluation revealed purulent peritonitis with the omentum localized over the first part of the duodenum and in the vicinity of the gall bladder. On lifting off the omentum, the nasogastric tube was seen perforating and protruding out from the first part of the duodenum and impacting on to the gall bladder (Figure 1). The perforation was 2 cm in diameter (Figure 2). Laparoscopic intracorporeal suturing and knotting was done for closure of the perforation using three interrupted 2-0 absorbable (polyglactin 910) sutures. The bites were taken 1 cm from the edge of the ulcer. The middle suture was tied first, followed successively by the upper and lower sutures and this was reinforced by an onlay omental pedicle (Figures 3(a) and 3(b)). The integrity of the repair was confirmed by the “tire test” (air insufflation via the NG tube). Blood loss was minimal. The operating time was 90 minutes. The postoperative period was uneventful. Bowel sounds were evident from the 2nd postoperative day and the patient was started on oral fluids by the 3rd POD and discharged on the 10th POD. An upper GI endoscopy 5 weeks later confirmed that perforation had healed well. The patient had been on regular followup for up to 10 months.

## 3. Discussion

### 3.1. Nasoenteral Tubes and Duodenal Perforation

The insertion of a nasogastric tube is a common clinical procedure which is relatively simple and safe. Nevertheless, various unexpected and potentially lethal complications have been reported [5]. The reported complication rates are between 0.3% and 15%. Duodenal perforations due to nasoenteral tubes have been reported to occur in the pediatric patients. It is postulated that the peristaltic activity propels the tube along the relatively rigid duodenal loop. In adults, endoscopic guided duodenal tube (postpyloric feeding) placements are known to be associated with complications such as bleeding and duodenal perforation [6]. This is probably the first case in the literature to report duodenal perforation due to nasogastric tube (NGT) in an adult. In this patient the NGT would have migrated further down beyond the pylorus. The initial clinical suspicion was the probability of perforated stress ulcer. Stress ulcers are known to occur in critically ill patients. Perforation due to stress ulcers is rare, occurring in less than 1% of surgical ICU patients [7]. The other possibility could be that the NG tube would have just found its way out through an already perforated stress/peptic ulcer. In either case if it had remained there for a longer time, it could have possibly migrated into the gall bladder, thereby making the situation even more hazardous. In order to prevent such serious complications, various methods of confirming proper placement of the nasogastric tube have been described and studied [8].

### 3.2. Laparoscopic Repair of Duodenal Perforations

Laparoscopic repair of duodenal perforations has been studied extensively with respect to perforated duodenal ulcers. Various studies including the LAMA (*LAparoscopische MAagperforatie*) trial have shown that the laparoscopic repair of peptic ulcer perforations is feasible, safe and associated with lesser postoperative pain, lower median hospital stay, earlier return to normal activity, and better cosmesis [9, 10].

The commonly encountered duodenal ulcer perforations are 1 cm or smaller, and these perforations are the easiest to repair either by open or laparoscopic techniques when compared to larger perforations. The outcome in this subset is also better. However there has been some confusion regarding categorizing duodenal perforations based on their size. The term “giant perforations” has been randomly used by various authors to describe the size of perforations ranging anywhere between 0.5 cm to 2.5 cm. A more meaningful classification based on the size of perforations has been suggested by Gupta et al. [11]. Accordingly, duodenal perforations can be classified into three main groups: *small* perforations that are less than 1 cm in size, *large* perforations that have a size between 1 cm and 3 cm, and *giant* perforations that exceed 3 cm size.

Bertleff and Lange [1] in their review opined that in case of suspected perforated peptic ulcer, laparoscopy should be advocated as diagnostic and therapeutic tool as there is a notable difference in morbidity (14.35 in laparoscopic group versus 26.9% in open group) and mortality (3.6% versus 6.4%). The overall conversion rate is around 12.4% (range 0–28.5%). The most common reason for conversion was the size of the perforation (often >10 mm), but by using a pedicled omentoplasty, size of the perforation might not necessarily be a reason to convert any longer.

In the present case, laparoscopic primary suture closure followed by onlay pedicled omentoplasty was used to repair the large perforation. However certain aspects need mentioning. Here it is felt that the perforation was not due to a duodenal ulcer and that is probably the reason why the edges were not friable and the sutures could be easily placed without the risk of tearing or cutting through. Moreover this was a young patient (with no known previous major medical illness) and the surgical treatment was accomplished before 24 hours after the onset of symptoms. In the present case, the patient was already on antibiotics, and this would have reduced the local inflammation and sepsis thereby making the procedure simpler. Therefore it is not certain whether the same technique could be replicated in case of a large duodenal ulcer perforation with greatly inflamed and friable margins. In such cases it would probably make sense to use a laparoscopic omental plug or laparoscopic version of a Cellan-Jones repair (suturing pedicled omentum on top of perforation without primary suture closure) or even convert to an open procedure which would probably require resective gastroduodenal surgery based on the surgeon's decision at that time. The factors associated with adverse outcomes after peptic ulcer perforations include older age, associated major medical illness, perforations of >24 hours duration, and delay in surgery beyond >12 hours after onset of symptoms [12, 13]. The lack of these adverse factors probably worked in favor of this patient. Therefore at present there is no strong evidence to support the role of laparoscopic technique for closure of large duodenal perforations. The probable argument would be that even with open surgery larger ulcers are more difficult to repair and the outcomes in this subset are poorer. Laparoscopic suture repair is more technically demanding and hence various novel methods are being developed to replace sutures for perforation closure. Nevertheless this paper illustrates the possibility of safe laparoscopic repair of large duodenal perforation.

## 4. Conclusions

Duodenal perforation secondary to nasogastric tubes is a rare complication in adults. Increasing awareness of potential complications associated with the insertion and maintenance of nasogastric tubes will facilitate early diagnosis and treatment. Laparoscopy is useful in the diagnosis and treatment of duodenal perforations and may be feasible for repairing large duodenal perforations. However, further research is needed to confirm the true benefits of laparoscopic repair for large or giant duodenal perforations.
